# The difference between 2-hour post-challenge and fasting plasma glucose associates with the risk of cardiovascular disease in a normoglycemic population: the Tehran lipid and glucose study

**DOI:** 10.1186/s12986-024-00782-3

**Published:** 2024-02-22

**Authors:** Amir Abdi, Karim Kohansal, Davood Khalili, Fereidoun Azizi, Farzad Hadaegh

**Affiliations:** 1 Prevention of Metabolic Disorders Research Center, Research Institute for Endocrine Sciences, Shahid Beheshti University of Medical Sciences, Tehran, P.O. Box: 19395-4763, Iran; 2 Student Research Committee, School of Medicine, Tehran Medical Sciences, Islamic Azad University, Tehran, Iran; 3 Endocrine Research Center, Research Institute for Endocrine Sciences, Shahid Beheshti University of Medical Sciences, Tehran, Iran

**Keywords:** Normoglycemia, 2-hour post-challenge plasma glucose, Fasting plasma glucose, Cardiovascular disease

## Abstract

**Background:**

Elevated fasting plasma glucose (FPG) and 2-hour post-challenge glucose (2hPG) levels are known to be independent risk factors for cardiovascular disease (CVD). However, there is limited data on the association of the difference between these measures and the risk of CVD. This study aims to investigate this association in normoglycemic Iranian adults, particularly in those with low-normal FPG levels.

**Methods:**

This prospective cohort study included 4,594 30-65-year-old participants from the Tehran Lipid and Glucose Study. Using multivariable Cox proportional hazards regression models adjusting for age, sex, body mass index, hypertension, hypercholesterolemia, smoking, education level and FPG, hazard ratios (HRs) and 95% confidence intervals (95% CIs) were calculated for the association between 2hPG-FPG, both as continuous and categorical variables, and the CVD risk. Analyses of receiver operating characteristic curves were undertaken to determine the optimal 2hPG-FPG cut-off value.

**Results:**

During a median of 17.9 years of follow-up, 459 CVD events occurred. A one-unit increase in 2hPG-FPG was significantly associated with an elevated risk of cardiovascular disease in both normoglycemic (HR 1.10, 95% CI (1.01–1.19)) and low-normal FPG individuals (HR 1.16, 95% CI (1.04–1.30)); this association resisted adjustment for Homeostatic Model Assessment for Insulin Resistance (HOMA-IR) among normoglycemic individuals. However, those with 2hPG levels greater than FPG levels had a non-significant increased risk of incident CVD compared to those with 2hPG levels of less than or equal to FPG, with corresponding HR values of 1.18 (95% CI: 0.95–1.46) in normoglycemic and 1.32 (95% CI: 0.98–1.79) in low-normal FPG, respectively. For incident CVD, the optimal cut-off value for the 2hPG-FPG was found to be 1.06 mmol/L, which was applicable for both normoglycemic and low FPG populations; using this criterion, the corresponding risks for incident CVD were 1.36 (95% CI: 1.12–1.64) and 1.57 (95% CI: 1.22–2.03), respectively.

**Conclusions:**

The difference between 2hPG and FPG levels within the normoglycemic range is related to an increased risk of CVD, an issue that was independent of HOMA-IR. A cut-off point for 2hPG-FPG > 1.06 mmol/L may stratify persons at higher risk. These findings were particularly notable in those with low-normal FPG.

**Supplementary Information:**

The online version contains supplementary material available at 10.1186/s12986-024-00782-3.

## Background

Over the past decades, cardiovascular diseases (CVDs) have persistently remained the leading cause of global morbidity and mortality, with coronary heart disease (CHD) and stroke as the main contributors [1]. It is reported that around 80% of CVD mortality worldwide occurs in middle- to low-income countries, where cardiovascular risk factors continuously increase [2]. Iran, similar to other countries in the Eastern Mediterranean Region (EMR), is no exception in this regard and continues to experience an increase in the prevalence of CVD and the subsequent epidemiological and socioeconomic burden related to it [3]. The incidence rate of CVD events among the Tehran population was reported to be 11 per 1000 person-years [4].

The impact of hyperglycemia on the development of CVDs is well established [5, 6]. Elevated fasting plasma glucose (FPG) and 2-hour post-challenge glucose (2hPG) levels are also known to be independent risk factors for CVD [7, 8]. Although there have been studies comparing the predictive values of 2hPG and FPG for CVD [9, 10], there is limited data on the association of the difference between these measures and the risk of CVD. Also, many previous studies were conducted among participants with glucose intolerance status but not among the normoglycemic population [11–13]. Researchers of the DECODE study group reported that within the normoglycemic range, individuals whose 2hPG did not return to their FPG levels during the oral glucose tolerance test (OGTT) had an increased risk for CHD [14], ischemic stroke [14], and mortality [15]. Moreover, they also found that increasing difference between 2hPG and FPG levels (2hPG-FPG) was also associated with unfavorable outcomes.

In the current study, we investigated the association of difference between 2hPG and FPG, either as a continuous or categorical variable, with the subsequent incidence of CVD among normoglycemic Tehranian adults as well as among subgroup with low-normal FPG (i.e., FPG < 5 mmol/L [16]) population. Moreover, we aimed to determine an appropriate cut-off point for this difference in the prediction of CVD.

## Materials and methods

### Study design and population

The Tehran Lipid and Glucose Study (TLGS) is a population-based prospective cohort study that focuses on determining the incidence, prevalence, and risk factors of non-communicable diseases. The design and process of this study have been described in detail before [17]. The TLGS recruitment involved selecting individuals from an urban population in Tehran who were three years or older. This was done during two phases, the first between 1999 and 2002 when they recruited 15,005 people and the second between 2002 and 2005 with an additional 3550 recruits. These participants were then examined again every three years during four subsequent phases: phase three (2005–2008), phase four (2009–2011), phase five (2012–2015), and finally, phase six (2015–2018).

**Fig. 1:**
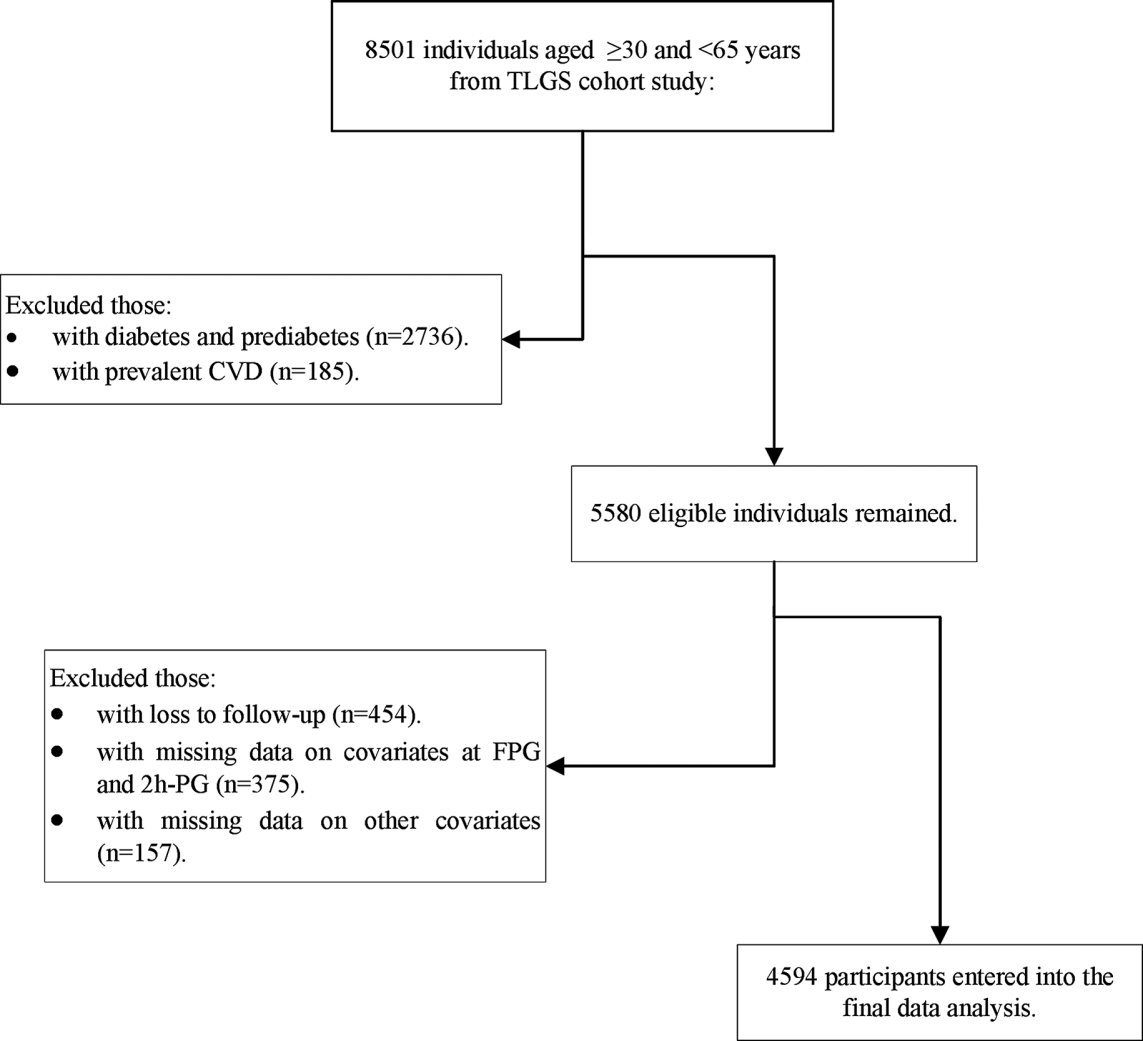
Study Flowchart, Tehran Lipid and Glucose Study, 2001-2018. TLGS: Tehran Lipid and Glucose Study; CVD: cardiovascular disease; FPG: fasting plasma glucose; 2hPG: 2-hour post-load plasma glucose

Figure 1 depicts the flow diagram of the study participants’ selection. In the present study, we included 8501 participants within the age range of ≥ 30 and < 65 years from the first (*n* = 7000) and second (*n* = 1501) phases. We excluded those with diabetes and pre-diabetes (*n* = 2736) and prevalent CVD at phases 1 and 2 (*n* = 185). From a total of 5580 eligible participants, we excluded those without any follow-up after baseline recruitment (*n* = 454), subjects with missing data for FPG and 2hPG measurements (*n* = 375), and also other study covariates (*n* = 157, considering overlap features between numbers). 4594 individuals with normoglycemia (FPG < 5.6 mmol/L and 2hPG < 7.8 mmol/L and not using glucose-lowering medication) remained in the study, and we followed them annually for the incidence of CVD until March 2018. The association between 2hPG-FPG with CVD events was also examined considering insulin resistance status in a subpopulation of TLGS participants with insulin data (*n* = 2432). This study was supervised and approved by the Institutional Review Board of the Research Institute for Endocrine Sciences (RIES) ethics committee and written informed consent from all participants was obtained.

### Clinical and laboratory measurements

Information on demographics, marital status, family history of T2DM, education, smoking habits, and medications using standardized interviewer-administered questionnaires was collected at baseline and repeated in each subsequent visit. Anthropometric measures were taken while the subjects were lightly dressed and without shoes. Weight was measured to the nearest kilogram using a digital scale. Height was measured to the nearest centimeter in a standing position with the shoulders in normal alignment using a tape measure. Body mass index (BMI) was calculated as weight (kg) divided by the square of height (m^2^). Waist circumference (WC) was measured to the nearest centimeter at the level of the umbilicus using an upstretched tape measure. For blood pressure measurement, the subjects first rested for fifteen minutes. Systolic and diastolic blood pressure (SBP and DBP, respectively) were measured twice on the right arm using a standardized mercury sphygmomanometer, and SBP and DBP were recorded as the mean of these readings. Participants fasted overnight for 12–14 h before taking a venous blood sample for FPG and lipid measurements. All participants who were not taking glucose-lowering medication underwent an oral glucose tolerance test by ingesting 82.5 g of glucose monohydrate (equivalent to 75 g of anhydrous glucose); a further blood sample was taken two hours later to assess 2-hour post-challenge glucose. The electrochemiluminescence immunoassay (ECLIA) technique, with the use of Roche Diagnostics kits and the Roche/Hitachi Cobas e-411 analyzer, was utilized to measure fasting serum insulin levels. The details of the clinical and laboratory measurements have been reported previously [17].

### Definition of terms

Subjects were considered normoglycemic if they met all of the following criteria according to the American Diabetes Association guidelines: (1) normal fasting glucose (NFG): had an FPG < 5.6 mmol/L, (2) normal glucose tolerance: had a 2hPG < 7.8 mmol/L, and (3) were not taking any glucose-lowering medication. Low-normal FPG was defined as an FPG < 5 mmol/L [16]. The difference between 2hPG and FPG as a continuous variable (2hPG-FPG) was calculated using the baseline values of these markers. We also categorized subjects into two groups according to the relative difference between their 2hPG and FPG levels: (1) subjects with 2hPG levels lower than or equal to their FPG levels (2hPG ≤ FPG); (2) subjects with 2hPG levels higher than their FPG levels (2hPG > FPG). HOMA-IR (Homeostatic Model Assessment for Insulin Resistance) was calculated as fasting insulin (IU/mL) × fasting glucose (mmol/L)]/22.5, as a surrogate of insulin resistance [18]. Homeostasis Model Assessment of Beta-cell function (HOMA-B) was defined as (Fasting insulin (mIU/l) x 20) / (Fasting glucose (mmol/L)– 3.5) as a surrogate of Beta-cell function [19]. Hypertension was defined as systolic blood pressure (SBP) ≥ 140 mmHg and/or diastolic blood pressure (DBP) ≥ 90 mmHg or the use of anti-hypertensive medication [20]. Hypercholesterolemia was defined by TC ≥ 5.17 mmol/L or the use of lipid-lowering medication [21]. Smoking status was classified as current smoker, ex-smoker (quit smoking at least one year before study entry), or never-smoker (reference group). Three groups of education status were also defined: (1) less than 6 years, (2) 6–12 years (reference group), and (3) more than 12 years, based on self-reported education level.

### Definition of the outcome

Incident CVD, the outcome of interest in the current study, was considered a composite of any fatal and non-fatal CHD and stroke events. CHD was classified into three types: angiographically proven CHD, definite myocardial infarction (MI) (diagnostic electrocardiography and biomarkers), and probable myocardial infarction (positive electrocardiograph findings plus cardiac symptoms or signs plus missing biomarkers or positive electrocardiograph findings plus equivocal biomarkers). Incident stroke was defined as all cases with confirmed and probable stroke as well as transient ischemic attack [22]. For cardiovascular events (i.e., ischemic heart disease (ICD-10 codes I20-I25), sudden cardiac death (I46.1), or stroke (ICD-10 codes I60-I69), TLGS employed ICD-10 criteria and AHA categorization [17]. Further details of the outcome measures and adjudication protocols.

have been published previously [23–25].

### Statistical analysis

**Fig. 2:**
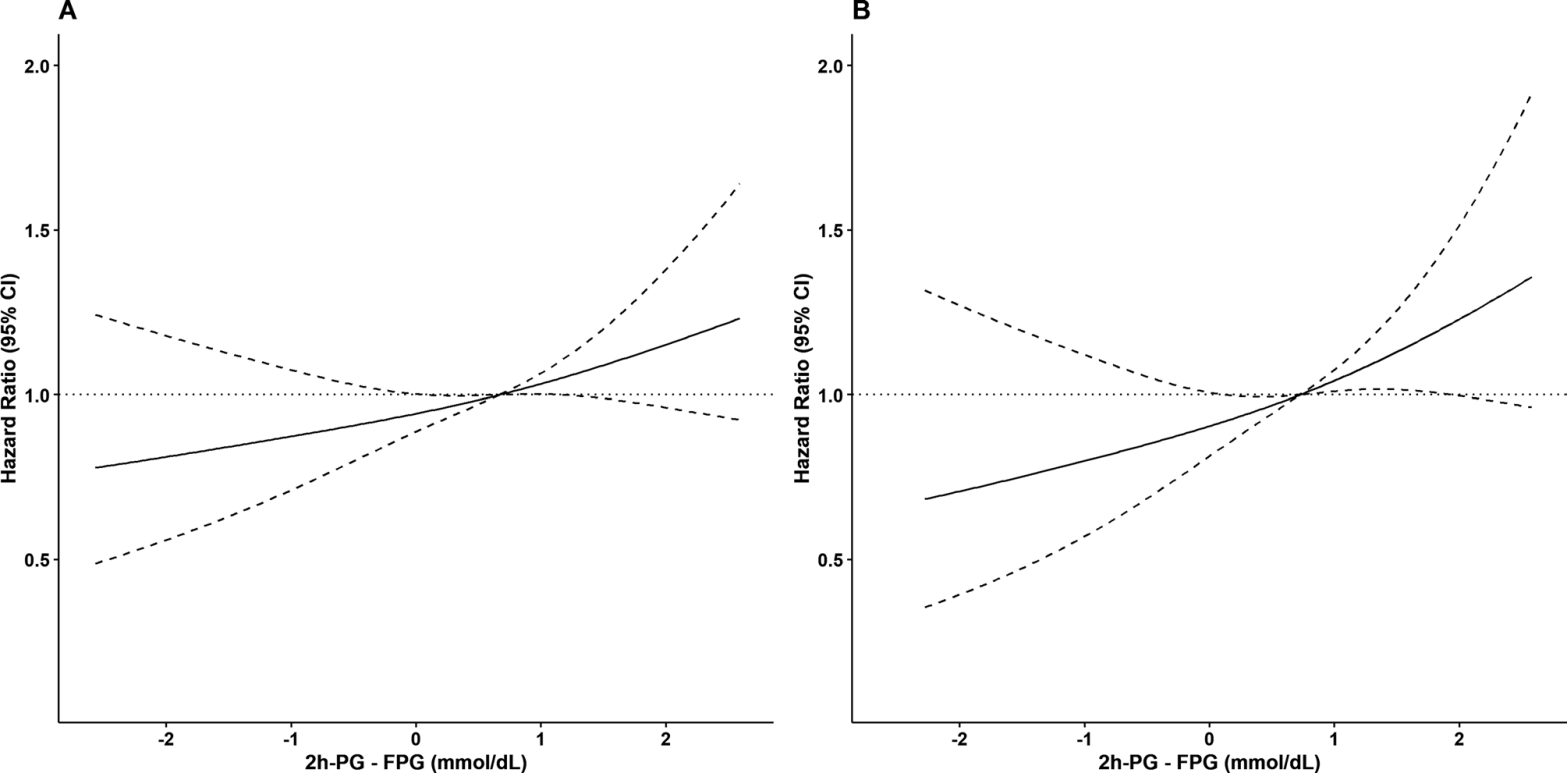
Restricted cubic spline curve for association (95% CI) of 2hPG-FPG with CVD events among study normoglycemic population (**A**) and low-FPG subpopulation (**B**). Low-normal FPG was defined as an FPG < 5 mmol/L. CVD: cardiovascular disease; FPG: fasting plasma glucose; 2hPG: 2-hour post-load plasma glucose

Continuous and categorical variables are summarized as mean (standard deviation (SD)) and number (percentage). Baseline characteristics of the study population were compared between subjects with 2hPG levels lower than or equal to their FPG levels (2hPG ≤ FPG) and subjects with 2hPG levels higher than their FPG levels (2hPG > FPG). We also compared baseline characteristics between participants and non-participants (including those without any follow-up after baseline recruitment or those with missing data). Comparisons were made using the student’s t-test for continuous variables and the chi-squared test for categorical variables. Crude incidence rate and 95% confidence interval (CI) per 1000 person-years were calculated for the whole population as well as the 2hPG ≤ FPG and 2hPG > FPG groups. Multivariable restricted cubic splines were used to account for a possible non-linear association between continuous exposure (2hPG-FPG) and incident CVD (Fig. 2).

Multivariable Cox proportional hazards regression models were used to calculate hazard ratios (HRs) and 95% CIs per unit (1 mmol/L) increase in difference between 2hPG and FPG and also for those with 2hPG > FPG compared to 2hPG ≤ FPG (as the reference). Five models were used: Model 1 was adjusted for age and gender. Model 2 was further adjusted for smoking status, education, BMI, hypertension, and hypercholesterolemia. Model 3, was further adjusted for baseline FPG levels. To assess the robustness of our results, we conducted Model 4 and Model 5 analyses by replicating Model 3 in sub-samples of the study population further adjusted for HOMA-IR and HOMA-B, respectively. We examined the variance inflation factor (VIF) of the variables included in the model to address the issue of multi-collinearity. We did not find evidence of multi-collinearity in the model, given the VIF of < 5 [26].

We also used the receiver operating characteristic (ROC) curve analysis to determine the optimum cut-off value for 2hPG-FPG and the incidence of CVD. The best cut-off point for 2hPG-FPG was assessed by the maximum value of the Youden index, which represented the maximum sum of sensitivity and specificity. The proportional hazard assumption in the Cox models was assessed with the Schoenfeld residual test; all proportionality assumptions were appropriate. All analyses were performed with R software (version 4.1.2), and two-sided *P* < 0.05 was considered statistically significant.

## Results

Among 4594 participants at the baseline, 57.1% were female, and the mean age was 42.1 years.

Table 1 presents the baseline characteristics of participants across two groups based on the difference between 2hPG and FPG (2hPG ≤ FPG and 2hPG > FPG). Participants in the 2hPG > FPG group had significantly higher mean values of age, BMI, SBP, DBP, 2hPG, 2hPG-FPG, and TC (*p* < 0.01 for all comparisons). Furthermore, the use of anti-hypertensive and lipid-lowering medications and the prevalence of hypertension and hypercholesterolemia were higher in the group with 2hPG > FPG, and subjects in the latter group were more current smokers and more educated. A total of 2760 individuals out of 4594 participants of the study population had low-normal FPG. The comparison of the baseline characteristics between the low- and high-normal (5 ≤ FPG < 5.6 mmol/L) is shown in Table S1. Generally, persons with high-normal FPG had more unfavorable baseline characteristics than their low-normal FPG counterparts. As expected, the 2hPG-FPG difference was higher among low-normal than high-normal group.

**Table 1:** Baseline characteristics of the study population, Tehran lipid and glucose study

Variables	Whole population (*n* = 4594)	2hPG ≤ FPG (*n* = 1352)	2hPG > FPG (*n* = 3242)	P-value
**Continuous variables**				
Age, year	42.1 (9.3)	40.8 (9.0)	42.6 (9.3)	< 0.01
**BMI, kg/m** ^**2**^	26.9 (4.4)	25.9 (4.4)	27.3 (4.3)	< 0.01
SBP, mmHg	115.1 (15.7)	113.0 (14.8)	115.9 (16.0)	< 0.01
DBP, mmHg	76.7 (10.2)	75.2 (10.1)	77.3 (10.2)	< 0.01
FPG, mmol/L	4.85 (0.38)	4.89 (0.36)	4.83 (0.38)	< 0.01
2hPG, mmol/L	5.47 (1.17)	4.10 (0.68)	6.04 (0.80)	< 0.01
2hPG-FPG, mmol/L	0.62 (1.16)	-0.78 (0.65)	1.21 (0.76)	< 0.01
TC, mmol/L	5.30 (1.12)	5.20 (1.12)	5.35 (1.11)	< 0.01
HDL-C, mmol/L	1.07 (0.28)	1.07 (0.29)	1.08 (0.28)	0.86
HOMA-IR*	1.70 (0.90)	1.56 (0.83)	1.75 (0.93)	< 0.01
HOMA-B*	127.06 (121.01)	133.77 (135.14)	110.43 (72.85)	< 0.01
**Categorical variables**				
Smoking (current, %)	786 (17.1)	308 (22.8)	478 (14.7)	< 0.01
Education (%)				< 0.01
< 6 years	722 (15.7)	227 (16.8)	495 (15.3)	
6–12 years	2567 (34.1)	805 (59.5)	1762 (54.3)	
> 12 years	1305 (28.4)	320 (23.7)	985 (30.4)	
Hypercholesterolemia (yes, %)	2379 (51.8)	634 (46.9)	1745 (53.8)	< 0.01
Hypertension (yes, %)	651 (14.1)	147 (10.9)	504 (15.5)	< 0.01
Anti-hypertensive drug use (yes, %)	157 (3.4)	28 (2.1)	129 (4.0)	< 0.01
Lipid-lowering drug use (yes, %)	81 (1.8)	16 (1.2)	65 (2.0)	0.07

BMI: body mass index; SBP: systolic blood pressure; DBP: diastolic blood pressure; FPG: fasting plasma glucose; 2hPG: 2-hour post-challenge glucose; HOMA-IR: Homeostatic Model Assessment for Insulin Resistance; HOMA-B: Homeostasis Model Assessment of Beta-cell function; TC: total cholesterol; HDL-C: high-density lipoprotein cholesterol; SD: standard deviation; IQR: interquartile range

Data are shown as mean (SD) for continuous variables or number (percent) for categorical variables

* In a subsample of the study population with insulin data, 2432 normoglycemic individuals

During a median follow-up of 17.9 years (IQR:13.7–18.5), 459 CVD events (164 women) occurred among the whole population; the corresponding value in the low-normal FPG subpopulation was 259 (103 women). The crud incidence rate of CVD was 6.41 (5.84–7.03) per 1000 person-years in the whole population and 6.00 (5.29–6.77) per 1000 person-years in the low-FPG subpopulation. Moreover, the group with a 2hPG > FPG had a higher incidence rate of CVD than the 2hPG ≤ FPG group; with crud incidence rates of 6.69 (5.99–7.44) and 5.76 (4.78–6.88) per 1000 person-years for 2hPG > FPG and 2hPG ≤ FPG groups, respectively. Moreover, the higher values of 2hPG-FPG were associated with a higher incidence rate of CVD. As shown in Table 2, the multivariate-adjusted HRs (95% CI) for incident CVD for the group with 2hPG > FPG compared to the group with 2hPG ≤ FPG were 1.18 (0.95–1.46) in the whole normoglycemic population; the corresponding value for those with FPG < 5 mmol/L was 1.32 (0.98–1.79) that tended to be significant (*p* = 0.068). Moreover, Table 2 shows the association between a unit (1 mmol/L) increase in 2hPG-FPG and incident CVD based on multivariate Cox regression analysis. As shown, in our analysis, a unit increase in the difference between concentration levels of 2hPG-FPG was significantly associated with increased CVD risk in both normoglycemic and low-normal FPG individuals, with the corresponding HR values of 1.10 (1.01–1.19) and 1.16 (1.04–1.30), respectively. In the analysis among participants with insulin data, after adjustments for HOMA-IR in model 4, 2hPG-FPG persisted to be associated with increased CVD risk in normoglycemic, but not low-normal FPG individuals. Moreover, adjustment for HOMA-B (Model 5) also did not affect the significant association of 2hPG-FPG and CVD in the whole normoglycemic, but the significance in the low-normal FPG population vanished. As an additional analysis, when we excluded the incident cases of prediabetes/T2DM during three years after baseline, the significant association between 2HPG-FPG difference and incident CVD disappeared (Table S3).

**Table 2:** Multivariable-adjusted hazard ratios for incidence of CVD, Tehran Lipid and Glucose Study, 2001–2018

	Model 1	p-value	Model 2	p-value	Model 3	p-value	Model 4	p-value	Model 5	p-value
**Whole normoglycemic population** (***N*** = 4594)										
2hPG-FPG (mmol/L)	1.10 (1.02–1.19)	0.019	1.09 (1.01–1.18)	0.026	1.10 (1.01–1.19)	0.023	1.14 (1.01–1.28)	0.029	1.15 (1.02–1.29)	0.023
2hPG > FPG	1.21 (0.98–1.49)	0.078	1.18 (0.95–1.46)	0.127	1.18 (0.95–1.46)	0.125	1.27 (0.93–1.72)	0.132	1.27 (0.94–1.73)	0.121
Low-normal FPG subpopulation* ***(N = 2760)***										
2hPG-FPG (mmol/L)	1.16 (1.04–1.29)	0.007	1.15 (1.03–1.28)	0.012	1.16 (1.04–1.30)	0.009	1.15 (0.98–1.35)	0.093	1.15 (0.98–1.35)	0.090
2hPG > FPG	1.37 (1.02–1.84)	0.039	1.32 (0.98–1.78)	0.071	1.32 (0.98–1.79)	0.068	1.21 (0.79–1.86)	0.381	1.21 (0.79–1.86)	0.375

Multivariable Cox proportional hazards regression models were used to calculate hazard ratios (HRs) and 95% CIs per unit (1 mmol/L) increase in difference between 2hPG and FPG and also for those with 2hPG > FPG compared to 2hPG ≤ FPG (as the reference)

Model 1: Adjusted for age + sex

Model 2: Model 1 + adjustments for BMI, HTN, hypercholesterolemia, Smoking, Education level

Model 3: Model 2 + further adjustments for FPG.

Model 4: Model 3 + further adjustments for HOMA-IR (in a subsample of the study population with insulin data, 2432 normoglycemic individuals and 1483 low-FPG individuals)

Model 5: Model 3 + further adjustments for HOMA-B (in a subsample of the study population with insulin data, 2432 normoglycemic individuals and 1483 low-FPG individuals)

CVD: cardiovascular disease- HR: hazard ratio- CI: confidence interval- FPG: fasting plasma glucose- 2hPG: 2-hour post-challenge glucose- BMI: body mass index- HTN: hypertension- HOMA-IR: Homeostatic Model Assessment for Insulin Resistance - HOMA-B: Homeostasis Model Assessment of Beta-cell function

*Low-normal FPG was defined as an FPG < 5 mmol/L

As shown in Table S2, in our data analysis, among normoglycemic individuals, whether below or above 5 mmol/L, 2hPG but not FPG level remained as a significant predictor.

**Fig. 3:**
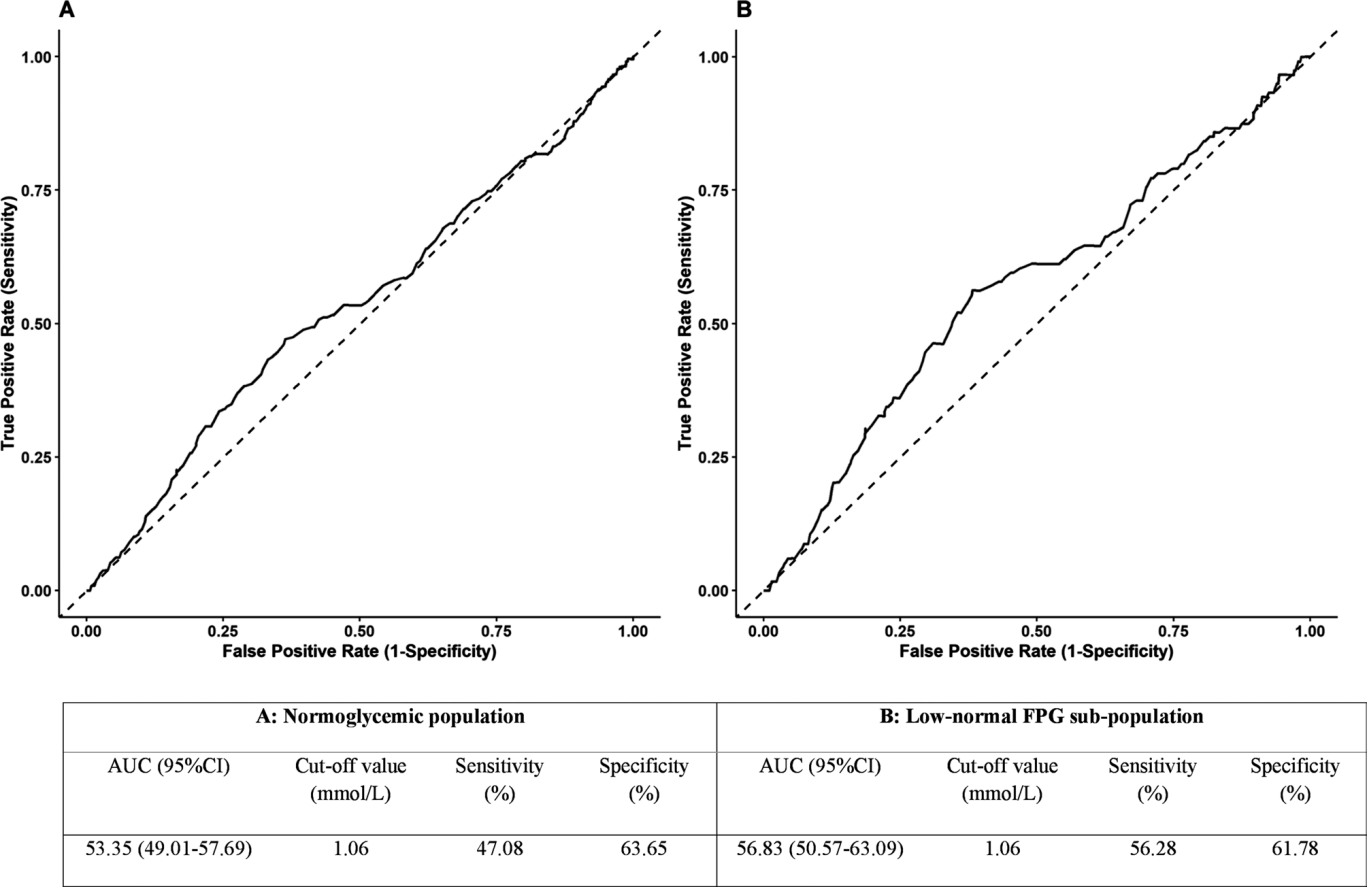
Receiver operating characteristic curves and cut of values of 2hPG-FPG for incident CVD among study normoglycemic population (**A**) and low-FPG subpopulation (**B**). Low-normal FPG was defined as an FPG < 5 mmol/L. CVD: cardiovascular disease; FPG: fasting plasma glucose; 2hPG: 2-hour post-load plasma glucose; AUC: area under the curve; CI: confidence interval

Additionally, in our ROC analysis (Fig. 3), in the whole population, the area under the curve (AUC) of the 2hPG-FPG for incident CVD was 53.35% (95% CI: 49.01-57.69%), and the calculated cut-off value for 2hPG-FPG was 1.06 mmol/L with a sensitivity of 47.08% and a specificity of 63.65%. The multivariable adjusted (model 3) hazard ratio for the derived cut-off point for the incident CVD was 1.36 (1.12–1.64, *p* = 0.002). In the low-normal FPG individuals, ROC analysis yielded an AUC of 56.83% (50.57-63.09%) with a 2hPG-FPG cut-off value of also 1.06 mmol/L, but with improved sensitivity and specificity of 56.28% and 61.78%, respectively. In these individuals, HR (model 3) of the 1.06 mmol/L cut-off point of 2hPG-FPG for the incident CVD was 1.57 (1.22–2.03, *p* < 0.001).

## Discussion

In the current study conducted in a population-based cohort in a normoglycemic population within the EMR region, we examined the association of the difference between 2hPG and FPG and incident CVD. We found that higher values of 2hPG-FPG were significantly associated with increased risk for CVD events with higher impact among those with low-normal FPG levels. Also, the discriminatory power (as demonstrated by AUC) of 2hPG-FPG for incident CVD among low-normal FPG was higher than normoglycemic individuals. The cut-off value of this difference was 1.06 mmol/L, which was associated with 36% and 57% risk for CVD events among normoglycemic and low-normal FPG populations, respectively.

In our study, when we categorized the population into two groups based on whether their 2hPG levels returned to their FPG levels or not, the group whose 2hPG concentrations did return or went above their FPG levels (2hPG > FPG) had a non-significant increased risk for incidence of CVD compared to the group who did (2hPG ≤ FPG). Succurro et al., in a study conducted on data from offspring subjects of T2DM patients, showed that the group with 2hPG > FPG had a worse cardiovascular risk profile (higher values of SBP, DBP, TG, and inflammatory markers) compared to 2hPG ≤ FPG participants [27]. In a study by the DECODE group [15], among a European population of 10,874 women and 12,566 men with normoglycemia, it was shown that within the normoglycemic range, elevated 2hPG levels were associated with an increased risk of mortality from CVD. Also, a one-unit increase in 2hPG-FPG was associated with more than 20% higher risk of CVD mortality. Later, in another study by the DECODE investigators [14], among participants of nine Swedish and Finnish cohorts, individuals with 2hPG and FPG values within the normoglycemic range whose 2hPG did not return to their FPG levels after an OGTT test had a worse cardiovascular profile and were at increased risk of stroke and CHD. Moreover, in line with our findings, the investigators observed that a 1-unit increase in 2hPG-FPG was significantly associated with the incident CVD. Recently, Wang et al. in a study among Chinese adults [28], found the impact of 2hPG on CVD events was more prominent among healthy individuals (i.e., those without hypertension, dyslipidemia, and obesity). Also, the investigators concluded that 2hPG, but not FPG had a significant impact on CVD incidence among those with FPG < 6.1 mmol/L. In the current study, we extended the previous research by showing that the impact of 2hPG-FPG as well as 2hPG was generally greater among those with FPG levels as low as 5mmol/L compared to those with FPG < 5.6 mmol/L. Also, as expected, after excluding the incident cases of prediabetes/diabetes, the significant association between 2hPG-FPG and CVD events, disappeared. Actually, we recently reported that among general Tehranian adults with prediabetes at the baseline, only those who converted to diabetes had a higher risk of developing CVD and persistent prediabetes during about three years was not associated with a higher risk, compared with those who reverted to normoglycemia [29].

We also examined the impact of HOMA-IR/HOMA-B on the association between 2hPG-FPG and cardiovascular events. Controlling for these indicators of insulin resistance and impaired insulin secretion did not change the association between 2hPG-FPG with CVD events among normoglycemic individuals; a similar effect size was also found among those with low-normal FPG, that did not reach the significant level due to the limited number of events. The hypothesis is that high post-load glucose levels in individuals with normal fasting and post-load glucose are associated with increased cardiovascular disease risk due to the pro-inflammatory state, regardless of insulin resistance and impaired insulin secretion. Studies have shown a relationship between fasting plasma glucose and coronary heart disease, possibly due to an enhanced pro-inflammatory state [30]. Moreover, the role of inflammation in the development of atherosclerosis and cardiovascular diseases independent of traditional CVD risk factors was addressed in many studies [31].

In the current study, we aimed to find the appropriate cutoff of 2hPG-FPG for defining those at risk of CVD events. Accordingly, the cut-off points of > 1.06 mmol/L (calculated using Youden’s index), particularly among those with FPG < 5 mmol/L, were associated with about 57% risk of CVD events. The derived cut-off points of the current study were very similar to those that stratify the Coronary Artery Risk Development in Young Adults (CARDIA) study participants at risk of developing T2DM, i.e., > 0.9 mmol/L [32].

Our study has some notable strengths, including its prospective population-based design, large sample size, and relatively long follow-up period. Moreover, to our knowledge, this is the first study attempting to determine a cut-off value for the association between 2hPG-FPG and CVD events. However, there are limitations; first, the current study was conducted among low-risk normoglycemic Tehranian participants who had a low incidence rate of CVD events compared with the general population [4]. Hence, the about 20 and 30% non-significant increased risk of CVD events for 2hPG > FPG compared to 2hPG ≤ FPG among whole and low-normal FPG population, respectively, could be attributed to the limited number of events among these individuals. Second, our study population was recruited from the metropolitan city of Tehran; hence the results may not be generalizable to rural zones. Second, there was a lack of data on glycosylated hemoglobin (HbA1c); however, the probability for misclassification in terms of glucose tolerance status was very low using both indicators of FPG and 2hPG. Third, as per the TLGS protocol we had only measured 2hPG and no data were available regarding other plasma glucose parameters and patterns during OGTT (i.e. 30 min and 1-hour post-load glucose tolerance test). Lastly, residual confounders such as dietary information may not have been taken into account.

## Conclusions

In conclusion, our study demonstrates that in a population-based study conducted in EMR with a high burden of CVD, the greater difference between 2-hour post-challenge glucose (2hPG) and fasting plasma glucose (FPG) (2hPG-FPG), is associated with the risk of CVD in individuals with normal glucose levels; the issue that was independent of insulin resistance status. Notably, this association was even greater in those with FPG < 5 mmol/L. Additionally, we found that a 2hPG-FPG of > 1 mmol/L was associated with a higher risk of CVD during more than a decade follow-up; the value that was also captured the risk of diabetes among normoglycemic Americans [32]. Hence future randomized controlled trials (RCTs) might address the role of lifestyle interventions in reducing this difference and possibly attenuating the risk of CVD events.

### Electronic supplementary material

Below is the link to the electronic supplementary material.

Supplementary Material: Table S1. Baseline characteristics of TLGS study population by respondents vs. non-respondents; Table S2. Multivariable-adjusted hazard ratios for incidence of CVD, Tehran Lipid and Glucose Study, 2001-2018

## Data Availability

The datasets used to reach the findings of the study are available from the corresponding author upon reasonable request.

## Abbreviations

AUC: area under the curve
BMI: body mass index
BP: blood pressure
CHD: coronary heart disease
CI: confidence interval
CV: coefficient of variation
CVD: cardiovascular disease
DBP: diastolic blood pressure
ECLIA: electrochemiluminescence immunoassay
EMR: Eastern Mediterranean Region
FPG: fasting plasma glucose
HOMA-B: Homeostasis Model Assessment of Beta-cell function
HOMA-IR: Homeostatic Model Assessment for Insulin Resistance
HR: hazard ratio
IFG: impaired fasting glucose
IGT: impaired glucose tolerance
IR: insulin resistance
MI: myocardial infarction
NFG: normal fasting glucose
OGTT: oral glucose tolerance test
RCT: randomized controlled trial
RIES: Research Institute for Endocrine Sciences
ROC: receiver operating characteristic
SBP: systolic blood pressure
SD: standard deviation
TC: total cholesterol
TLGS: Tehran lipid and glucose study
TG: triglycerides
T2DM: type 2 diabetes mellitus
VIF: variance inflation factor
WC: Waist circumference
2hPG: 2-hour post-challenge glucose
