# Supplementary material for: The difference between 2-hour post-challenge and fasting plasma glucose associates with the risk of cardiovascular disease in a normoglycemic population: the Tehran lipid and glucose study
Source: Nutr Metab (Lond). 2024 Feb 22;21:10. doi: 10.1186/s12986-024-00782-3 (PMC10882815; doi:10.1186/s12986-024-00782-3)
Supplement: Supplementary file 1 — Supplementary Material: Table S1. Baseline characteristics of TLGS study population by respondents vs. non-respondents; Table S2. Multivariable-adjusted hazard ratios for incidence of CVD, Tehran Lipid and Glucose Study, 2001-2018 [file 12986_2024_782_MOESM1_ESM.docx]

**Supplementary Materials**

| **Table S1 - Baseline characteristics of the Low-normal (FPG<5 mmol/L) and High-normal FPG (5** **≤ FPG<5.6** **mmol/L) population, Tehran Lipid and Glucose Study** | | | | |
| --- | --- | --- | --- | --- |
| **Variables** |  | Low normal FPG  (n=2760) | High normal FPG  (n=1834) | P-value |
| **Continuous variables** |  |  |  |  |
| Age, year |  | 41.4 (9.3) | 43.1 (9.4) | <0.01 |
| BMI, kg/m^2^ |  | 26.6 (4.4) | 27.4 (4.3) | <0.01 |
| SBP, mmHg |  | 113.8 (15.5) | 117.0 (15.9) | <0.01 |
| DBP, mmHg |  | 76.0 (10.3) | 77.8 (10.0) | <0.01 |
| FPG, mmol/L |  | 4.61 (0.27) | 5.22 (0.15) | <0.01 |
| 2hPG, mmol/L |  | 5.33 (1.14) | 5.68 (1.18) | <0.01 |
| 2hPG-FPG, mmol/L |  | 0.72 (1.14) | 0.46 (1.18) | <0.01 |
| TC, mmol/L |  | 5.23 (1.09) | 5.42 (1.14) | <0.01 |
| HDL-C, mmol/L |  | 1.09 (0.28) | 1.05 (0.28) | 0.86 |
| HOMA-IR* |  | 1.53 (0.79) | 1.96 (1.00) | <0.01 |
| HOMA-B* |  | 145.33 (146.84) | 98.49 (50.03) | <0.01 |
| **Categorical variables** |  |  |  |  |
| Smoking (current, %) |  | 478 (17.3) | 308 (16.8) | 0.67 |
| Education (%) |  |  |  | 0.02 |
| < 6 years |  | 437 (15.8) | 285 (15.5) |  |
| 6-12 years |  | 1581 (57.3) | 986 (53.8) |  |
| > 12 years |  | 742 (26.9) | 563 (30.7) |  |
| Hypercholesterolemia (yes, %) |  | 1368 (49.6) | 1011 (55.1) | <0.01 |
| Hypertension (yes, %) |  | 352 (12.8) | 299 (16.3) | <0.01 |
| Anti-hypertensive drug use (yes, %) |  | 84 (3.0) | 73 (4.0) | 0.10 |
| Lipid-lowering drug use (yes, %) |  | 49 (1.8) | 32 (1.7) | <0.01 |
| BMI: body mass index- SBP: systolic blood pressure- DBP: diastolic blood pressure- FPG: fasting plasma glucose- 2hPG: 2-hour post-challenge glucose- HOMA-IR: Homeostatic Model Assessment for Insulin Resistance- HOMA-B: Homeostasis Model Assessment of Beta-cell function- TC: total cholesterol- HDL-C: high-density lipoprotein cholesterol- SD: standard deviation- IQR: interquartile range.  Data are shown as mean (SD) for continuous variables or number (percent) for categorical variables.  * In a subsample of the study population with insulin data, 2432 normoglycemic individuals | | | | |

| Table S2 - Multivariable-adjusted hazard ratios for incidence of CVD, Tehran Lipid and Glucose Study, 2001-2018 | | | | | | |
| --- | --- | --- | --- | --- | --- | --- |
|  | **Model 1** | p-value | **Model 2** | p-value | **Model 3** | p-value |
| Whole normoglycemic population (N = 4594) |  |  |  |  |  |  |
| FPG (mmol/L) | 1.09 (0.85-1.38) | 0.506 | 1.01 (0.79-1.29) | 0.920 | 0.96 (0.75-1.23) | 0.761 |
| 2hPG (mmol/L) | 1.11 (1.02-1.20) | 0.011 | 1.09 (1.01-1.18) | 0.024 | 1.10 (1.01-1.19) | 0.023 |
| Low-normal FPG subpopulation* (N = 2760) |  |  |  |  |  |  |
| FPG (mmol/L) | 1.39 (0.87-2.20) | 0.164 | 1.20 (0.75-1.92) | 0.440 | 1.11 (0.70-1.78) | 0.658 |
| 2hPG (mmol/L) | 1.18 (1.06-1.32) | 0.002 | 1.16 (1.04-1.30) | 0.007 | 1.16 (1.04-1.30) | 0.008 |
| Multivariable Cox proportional hazards regression models were used to calculate hazard ratios (HRs) and 95% CIs per unit (1 mmol/L) increase in 2hPG and FPG.  *Low-normal FPG was defined as an FPG<5 mmol/L.  Model 1: Adjusted for age + sex  Model 2: Model 1 + adjustments for BMI, HTN, hypercholesterolemia, Smoking, Education level  Model 3: Model 2 + further adjustments for FPG/2hPG.  CVD: cardiovascular disease; HR: hazard ratio; CI: confidence interval; FPG: fasting plasma glucose; 2hPG: 2-hour post-challenge glucose; BMI: body mass index; HTN: hypertension. | | | | | | |

| **Table S4 - Multivariable-adjusted hazard ratios for incidence of CVD, Tehran Lipid and Glucose Study after exclusion incident diabetes and prediabetes within first three years of the follow-up period, 2001-2018** | | | | | | | | | | |
| --- | --- | --- | --- | --- | --- | --- | --- | --- | --- | --- |
|  | Model 1 | p-value | Model 2 | p-value | Model 3 | p-value | Model 4 | p-value | Model 5 | p-value |
| **Whole normoglycemic population (N =2562)** |  |  |  |  |  |  |  |  |  |  |
| 2hPG-FPG (mmol/L) | 1.04 (0.93-1.15) | 0.495 | 1.05 (0.94-1.16) | 0.397 | 1.04 (0.94-1.16) | 0.458 | 1.10 (0.95-1.27) | 0.221 | 1.09 (0.94-1.26) | 0.249 |
| 2hPG > FPG* | 1.11 (0.84-1.46) | 0.470 | 1.13 (0.85-1.48) | 0.400 | 1.12 (0.85-1.47) | 0.433 | 1.28 (0.87-1.89) | 0.206 | 1.27 (0.86-1.87) | 0.225 |
| **Low-normal FPG subpopulation (N = 1642)** |  |  |  |  |  |  |  |  |  |  |
| 2hPG-FPG (mmol/L) | 1.11 (0.97-1.27) | 0.138 | 1.11 (0.97-1.28) | 0.135 | 1.11 (0.97-1.28) | 0.121 | 1.15 (0.95-1.39) | 0.166 | 1.15 (0.95-1.39) | 0.158 |
| 2hPG > FPG* | 1.34 (0.93-1.92) | 0.118 | 1.37 (0.95-1.98) | 0.096 | 1.37 (0.95-1.99) | 0.091 | 1.40 (0.83-2.37) | 0.213 | 1.40 (0.83-2.37) | 0.207 |
| Multivariable Cox proportional hazards regression models were used to calculate hazard ratios (HRs) and 95% CIs per unit (1 mmol/L) increase in difference between 2hPG and FPG and also for those with 2hPG >FPG compared to 2hPG ≤FPG (as the reference).  *Low-normal FPG was defined as an FPG<5 mmol/L.  Model 1: Adjusted for age + sex  Model 2: Model 1 + adjustments for BMI, HTN, hypercholesterolemia, Smoking, Education level  Model 3: Model 2 + further adjustments for FPG.  Model 4: Model 3 + further adjustments for HOMA-IR (in a subsample of the study population with insulin data, 2432 normoglycemic individuals and 1483 low-FPG individuals).  Model 5: Model 3 + further adjustments for HOMA-B (in a subsample of the study population with insulin data, 2432 normoglycemic individuals and 1483 low-FPG individuals).  CVD: cardiovascular disease- HR: hazard ratio- CI: confidence interval- FPG: fasting plasma glucose- 2hPG: 2-hour post-challenge glucose- BMI: body mass index- HTN: hypertension- HOMA-IR: Homeostatic Model Assessment for Insulin Resistance - HOMA-B: Homeostasis Model Assessment of Beta-cell function. | | | | | | | | | | |
